# Effectiveness of COVID-19 Convalescent Plasma Infusion Within 48 Hours of Hospitalization With SARS-CoV-2 Infection

**DOI:** 10.7759/cureus.16746

**Published:** 2021-07-30

**Authors:** Natalia Lattanzio, Cristina Acosta-Diaz, Ricardo J Villasmil, Zachary Kirkland, Caitlin Bass, Sage Yenari, Jorge Conte, Kevin Dawkins, Tamela Fonseca, Cindy Grimes, Angie Stewart, Mary E Geary, Harold Vore, Karen Hamad, Wilhelmine Wiese-Rometsch, James Fiorica, Manuel Gordillo, Roberto Mercado, Kirk Voelker

**Affiliations:** 1 Internal Medicine, Sarasota Memorial Hospital, Florida State University College of Medicine, Sarasota, USA; 2 Internal Medicine, Sarasota Memorial Hospital, Sarasota, USA; 3 Medicine, Sarasota Memorial Hospital, Sarasota, USA; 4 Clinical Research, Sarasota Memorial Hospital, Sarasota, USA; 5 Clinical Database Administration, Sarasota Memorial Hospital, Sarasota, USA; 6 Quality Improvement, Sarasota Memorial Hospital, Sarasota, USA; 7 Pathology, Sarasota Memorial Hospital, Sarasota, USA; 8 Oncology, Sarasota Memorial Hospital, Sarasota, USA; 9 Infectious Disease, Sarasota Memorial Hospital, Sarasota, USA; 10 Pulmonology and Critical Care, Sarasota Memorial Hospital, Sarasota, USA

**Keywords:** covid-19, convalescent plasma, sars-cov-2, discharge to home, infusion

## Abstract

On January 30, 2020, the World Health Organization (WHO) declared the severe acute respiratory syndrome coronavirus 2 (SARS-CoV-2) pandemic a worldwide emergency. Worldwide there have been 170 million cases of the resulting disease coronavirus 2019 (COVID-19), of those, 3.53 million have resulted in death. The Food and Drug Administration (FDA) with Mayo Clinic as the lead institution authorized COVID-19 convalescent plasma (CCP) for treatment of SARS-CoV-2 infection. Effective therapeutic window for CCP administration had yet to be defined. We addressed this gap by characterizing longitudinal biologic response and clinical outcomes of COVID-19 patients treated with CCP. Primary outcome was discharged to home/home health.

## Introduction

On January 30, 2020, the World Health Organization (WHO) declared the severe acute respiratory syndrome coronavirus 2 (SARS-CoV-2) pandemic a worldwide emergency. Worldwide there have been 114 million cases of the resulting disease coronavirus 2019 (COVID-19) of those, 2.54 million have resulted in death. At pandemic debut, available treatment was limited to supportive care as no approved therapy or vaccination was available. This treatment vacuum motivated the utilization of convalescent plasma infusion to complement the antibody response.

Passive immunization has been successfully used to treat infectious diseases since the 1890s [1,2]. Convalescent plasma (CP) was used to treat Spanish Influenza A (H1N1), severe acute respiratory syndrome in 2003 caused by SARS-associated coronavirus (SARS-CoV), and Influenza A pandemic in 2009 [3,4]. Underpinning CP treatment is that subjects recovering from viral infection marshalled an effective antibody response. And donated CP administered to an infected individual anticipating transfused antibodies would affect sufficient passive immunity to reduce symptoms and mortality risk.

Results from small case series conducted during the prior Middle East Respiratory Syndrome (MERS) and SARS-CoV documented CP to be safe, well-tolerated and promoted faster viral clearance, particularly when given early in the disease course [5]. The primary hypothesized mechanism of action of CP in COVID-19 pathogenesis involves antibody neutralization downregulating the hyperinflammatory response evoked by SARS-CoV-2 ribonucleic acid (RNA) [6,7]. Also, the thought was that transfused antibodies passively increased tissue repair decreasing and/or obviating complications and progression to death [8-11]. Additional preliminary clinical evidence suggested that CP might benefit individuals with SARS-CoV-2 infection and symptom onset suggestive of COVID-19.

The Food and Drug Association (FDA) with Mayo Clinic as the lead institution authorized COVID-19 convalescent plasma (CCP) for treatment of CoV-2 infection. Effective therapeutic window for CCP administration had yet to be defined. We addressed this gap by characterizing longitudinal biologic response and clinical outcomes of COVID-19 patients treated with CCP.

## Materials and methods

Study design and population** ** 

This retrospective longitudinal study analyzed electronic medical record data, including but not limited to characteristics and laboratory test findings from 197 patients consecutively admitted between March 28 and August 5, 2020. Among those, 92 and 105, respectively, received CCP infusion within 48h of versus 48h after hospitalization. Primary outcome was discharged to home/home health and secondary outcome was longitudinal CRP levels post CCP infusion. 

Sarasota Memorial Hospital Institutional Review Board authorized consenting COVID-19 inpatients to participate in the national CCP protocol. Written informed consent was obtained from every CCP recipient or their legal guardian. Internal medicine resident physicians identified, contacted, and facilitated logistical pathways for CCP donation in collaboration with the community blood bank.

Enrolled patients were at least 18 years old with laboratory confirmed SARS-CoV-2 infection admitted for treatment of severe or life-threatening COVID-19. Severe disease was defined as the presence of at least one of the following characteristics: dyspnea, respiratory rate of 30 breaths per minute or more, oxygen saturation (SpO2) equal to or less than 93%, partial pressure of arterial oxygen to fraction of inspired oxygen less than 300 or development of lung infiltrates with more than 50% involvement within 24-48 hours (h). Life-threatening disease was defined as the development of at least one of the following: respiratory failure, septic shock or multiple organ dysfunction or failure.

Data analysis

Analyses contrasted patients who underwent CCP infusion within or more than 48h after admission. Primary outcome was discharged to home/home health. Continuous data summarized as median (interquartile range [IQR]) were compared using Kruskal-Wallis test or two-way analysis of variance (ANOVA). Discrete data were compared with Pearson’s chi-square test. Two-tailed p < 0.05 was significant.

## Results

The study included 197 patients consecutively admitted between March 28, 2020 and August 5, 2020. Of those, 92 received CCP infusion within 48h and 105 received CCP infusion after 48h. Median age in the group that received CCP within 48h was 67 years and 63 years in the group who received CCP after 48h. Of the 197 patients, 57% were male. Intergroup comorbidities were distributed similarly among both groups for the exception of body mass index (BMI) (32.7 (27-40)) vs. (29.4 (26-37) kg/m^2^, p < 0.0001) (Table 1). Distribution of COVID-19 directed pharmacologic treatment was also similar amongst both groups.

**Table 1:** Baseline characteristics of consecutive patients with COVID-19. IQR: interquartile range; N: number; BMI: body mass index; CRP: C-reactive protein; s: seconds; SpO2: oxygen saturation; aPTT: activated partial thromboplastin time.

Characteristics	Convalescent plasma > 48 hours	Convalescent plasma < 48 hours
	(N = 105)	(N = 92)
Age, median (IQR),	67 (59-78)	63 (50-71)
Sex, No. (%)		
Male	59 (56)	54 (59)
Female	46 (44)	38 (41)
Race, No. (%)		
White	66 (63)	57 (62)
Black	15 (14)	9 (10)
Other	24 (23)	26 (28)
Anthropometrics, median (IQR)		
Height (cm)	166 (160-177)	168 (161-178)
Weight (kg)	87.4 (70.6-104.0)	97.3 (78.0-112.2)
BMI (kg/m^2^)	29.4 (26-37)	32.7 (27-40)
Vital signs, median (IQR)		
Body temperature (°F)	98.3 (98.0-98.9)	98.5 (98.1-99.0)
Inspired O_2 _(%)	70 (51-90)	80 (58-100)
SpO_2_ (%)	95 (93-97)	95 (93-97)
Respiratory rate, /min	19 (18-21)	20 (19-21)
Heart rate, /min	77 (66-86)	80 (70-93)
Systolic blood pressure, mmHg	129 (117-143)	129 (117-140)
Coexisting diseases, No. (%)		
Hypertension	60 (59)	38 (43)
Diabetes	43 (41)	28 (30)
Obesity	43 (41)	45 (51)
Kidney disease	19 (19)	18 (20)
Depression	16 (16)	12 (14)
Neurological disease	14 (14)	13 (15)
Congestive heart failure	11(11)	9 (10)
COVID-19 pharmacotherapy		
Dexamethasone	86 (82)	73 (79)
Remdesivir	65 (62)	64 (70)
Azithromycin	17 (16)	21 (23)
Tocilizumab	13 (12)	12 (13)
Hydroxychloroquine	7 (7)	4 (4)
Laboratory tests, median (IQR)		
Inflammatory biomarkers		
CRP, mg/dL	9.3 (4.3-15.6)	11.90 (7.03-15.90)
Ferritin, ng/mL	592 (302-1165)	657 (259-1290)
Lactate dehydrogenase, U/L	326 (274-428)	366 (301-469)
Procalcitonin, ng/mL	0.12 (0.05-0.98)	0.14 (0.05-0.59)
Triglycerides, mg/dL	200(118-297)	161 (99-236)
Liver and kidney function		
Aminotransferase, U/L		
Alanine	38 (22-60)	39 (23-68)
Aspartate	42 (29-61)	41 (31-68)
Creatinine, mg/dL	1.1 (0.8-1.5)	1.1 (0.9-1.4)
Complete blood cells count		
White blood cells, /mL	6.80 (5.20-10.10)	7.05 (5.35-9.08)
Neutrophil, /mL	5.18 (3.70-7.95)	5.49 (3.90-7.20)
Lymphocyte, /mL	19 (12-28)	18 (12-25)
Hemoglobin, gm/dL	13.2 (11.3-14.7)	12.1 (14.2-15.4)
Coagulation profiles		
D-dimer, mg/mL	1.00 (0.72-2.04)	1.02 (0.66-2.01)
Prothrombin time, s	11.3 (10.8-12.0)	11.4 (10.7-12.3)
aPTT, s	30.2 (27.3-32.9)	30.8 (26.4-32.9)
Cardiac biomarkers		
Creatine Kinase Total, U/L	131 (63-271)	132 (70-381)
Troponin I, ng/mL	0.02 (0.02-0.13)	0.02 (0.02-0.06)
Pro-BNP, pg/mL	1071 (219-3141)	225 (58-3004)

**Figure 1:**
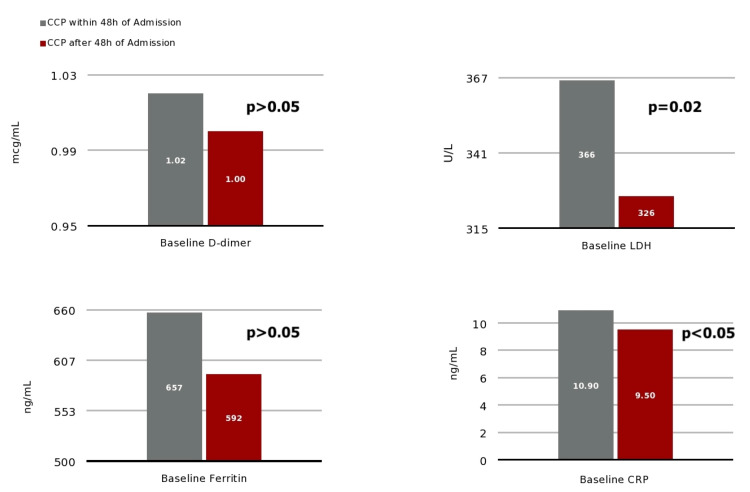
Baseline values for D-dimer, LDH, ferritin and CRP, respectively, for patients who received CCP within 48h of admission vs. 48h after admission. LDH: lactate dehydrogenase; CRP: C-reactive protein; CCP: COVID-19 convalescent plasma.

Initial admission vital signs and laboratory test results were not different between groups (p>.05) including temperature (98.4 (98.0-99.0) °F); SpO2 (95, (93-97) %); C-reactive protein (CRP) (2.6 (0.3-2.7) mg/dL); D-dimer (1.01 (0.69-2.03) mg/L); and ferritin (624 (281-1228) ng/mL) (Figure 1).

**Figure 2:**
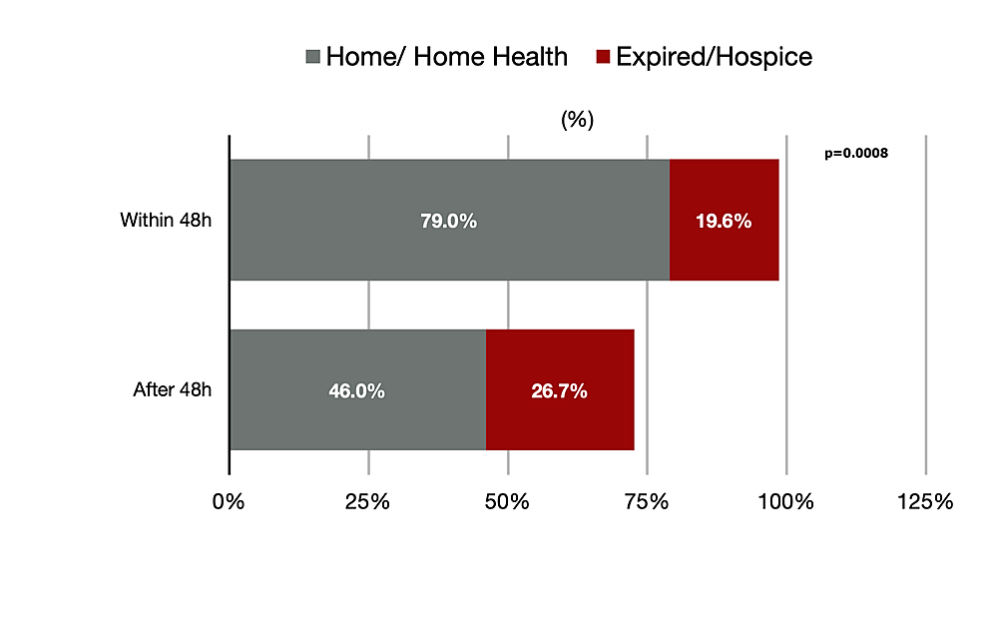
Percentage of patients discharged home/home health vs. expired/hospice.

Time from admission to CCP infusion was 32.4 (22.0-41.2) vs. 81.7 (61.3-130.0) hours who respectively received CCP infusion within vs after 48h, p < 0.0001. Admission lactate dehydrogenase (LDH) was (366 (301-469) U/L) vs. (326 (274-428) U/L, p = 0.02) (Figure 2).

**Figure 3:**
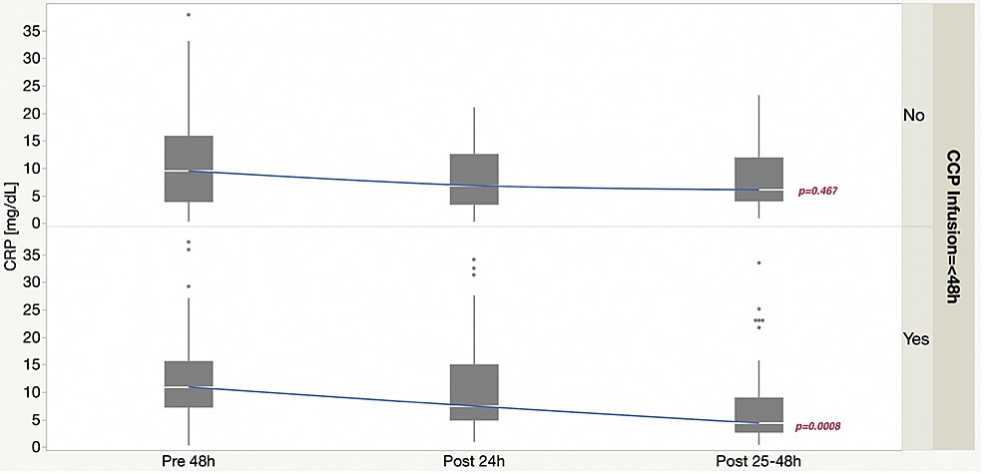
Longitudinal CRP levels within 48h, 24h post- and 25-48 post-CCP infusion, respectively. Two-way ANOVA demonstrated greater overall reduction in CRP when CCP was infused within 48h, p = 0.16. CRP: C-reactive protein; CCP: COVID-19 convalescent plasma; ANOVA: analysis of variance.

Patients intubated included 25/92 (27%) vs. 29/105 (28%), p = 0.34. Days of mechanical ventilation were 8.0 (5.0-11.8) vs. 11.3 (4.5-17.9), p = 0.16. Hospital length of stay was 8.5 (4.9-15.2) vs. 13.0 (6.5-18.9) days, p = 0.03 (Table 2). Longitudinal CRP levels within 48h pre-, 24h post- and 25-48 post-CCP infusion, respectively, were (10.9 (7.1-16.7) vs. 9.5 (3.9-15.9)) (p = 0.12), 7.5 (4.9-15.1) vs. 6.9 (3.3-12.6) (p = 0.20), and 4.4 (2.7-9.0) vs. 6.1 (4.0-12.0), (p = 0.02) (Figure 3).

**Table 2:** Outcomes. ICU: intensive care unit; LOS: length of stay; N = number; IQR: interquartile range; DC: discharge.

Clinical outcomes	Convalescent plasma > 48h	Convalescent plasma < 48h
	(N = 105)	(N = 92)
ICU admission, No. (%)	40 (38)	34 (37)
ICU days, median (IQR)	9.1 (3.0-16.6)	7.8 (2.9-12.1)
Intubation, No. (%)	30 (29)	23 (25)
Ventilation days, median (IQR)	11.3 (4.5-17.9)	8.0 (5.0-11.8)
Survivor not Hospice LOS	11.0 (5.9-18.7)	7.0 (4.7-12.9)
Overall LOS, median (IQR)	13.0 (6.5-18.9)	8.5 (4.9-15.2)
Discharge to home, No. (%)	51 (49)	67 (73)
Expired or Hospice DC, No. (%)	28 (26.7)	18 (19.6)

Primary outcome was discharged to home/home health which was 73/92 (79%) vs. 49/105 (46%), p = 0.005.

## Discussion

Between March 28 and August 5, 2020, Sarasota Memorial Hospital participated in the Mayo Clinic-led national FDA expanded access program providing access to convalescent plasma protocol. Throughout this period, data from 197 participants were analyzed and contrasted among those who received CCP infusion within or more than 48h after admission with a confirmed COVID-19 infection. We observed a post-CCP reduction in C-reactive protein, lower hospital length of stay and increase in discharge directly from hospital to home/home health in patients who received CCP within 48h of admission rather than later in hospitalization. Moreover, earlier CCP treatment resulted in 7% fewer patients who died or were discharged to hospice.

In December 2019, a new member of Coronaviridae family, SARS-CoV-2 was detected in Wuhan, China primarily manifesting as a respiratory illness [3]. Since then, multiple studies have been conducted exploring varied treatments for this lethal disease which has led to nearly 114 million of positive COVID-19 cases and 500,000 deaths in the United States. Presented with state of emergency accompanied by no available treatment and scarce medical resources; multiple centers around the world instituted CCP in a relatively timely manner previously shown effective during the SARS-CoV and Spanish Flu pandemic [3].

Initial evidence demonstrated CCP to be most beneficial when administered soon after SARS-CoV-2 infection. A randomized, double-blinded, placebo-controlled trial evaluated disease progression in 80 patients who received convalescent plasma within 72h after onset of mild COVID-19 symptoms vs. placebo [9]. Disease progression was advancement to severe respiratory disease defined as respiratory rate more than 30 breaths per min and/or arterialized blood oxygen saturation less than 93% breathing ambient air. Of the 80 patients, 31% of the patients who received placebo vs. 16% who received CCP progressed to severe respiratory disease [9]. Life-threatening respiratory disease was observed in only 5% of CCP patients vs. 12% in the placebo group. This study concluded that the administration of CCP to infected patients within 72h after the onset of symptoms reduce the risk of progression to severe respiratory disease by 48% [9]. CCP has demonstrated benefit in the clearance of SARS-CoV-2 in individuals who are symptomatic with COVID-19, including immunocompromised patients [8,10,11,12]. A cohort study of 966 patients with hematologic cancer and COVID-19 showed that after CP treatment, there was a significantly improved 30-day mortality [13].

CCP transfusion is associated with a reduction in inflammatory markers, such as CRP [8,10,14]. In a case series, 20 patients were treated with CCP compare to 20 controls matched with severe or life-threatening COVID-19 infection [15]. Results showed a marked reduction in CRP levels seven days after CCP infusion compared to control group. Discharges were similar amongst both groups, but mortality was higher in the control group. No patient died if they received CCP prior to seven days of hospitalization [15]. Another study reported lack of benefit when CCP was administered later (median of 21.5 days after diagnosis) for SARS-CoV-2, which supports that earlier treatment may be of critical importance [16]. Our longitudinal CRP observations corroborate and extend evidence favoring least “door to treatment” time. 

Our data should be interpreted with some caveats. We conducted a monocenter pragmatic investigation. CCP was administered before FDA required titer labeling. Therefore, we couldn’t establish if antibody titers in CCP transfused across patient groups were equivalently distributed. In addition, SARS-CoV-2 serologic testing was unreliable restricting the assessment of whether a patient exhibited an impaired humoral response.

## Conclusions

Our study was conducted while no anti-viral treatment was approved by the FDA for patients hospitalized with COVID-19. We evince CCP treatment within 48h of admission was associated a reduction in hyperinflammation and hospital length of stay in patients more obese with higher LDH levels with greater benefit for discharge to home/home health benefit and reduction in composite outcome of hospital mortality or discharge to hospice. Convalescent plasma has shown to be an effective treatment when given soon after SARS-CoV-2 infection. Unfortunately, in hospitalized patients with COVID-19, infusion of CCP late in the course of illness provides no observed benefit, as reported with other anti-viral agents.
